# The antineoplastic properties of FTY720: evidence for the repurposing of fingolimod

**DOI:** 10.1111/jcmm.12635

**Published:** 2015-07-14

**Authors:** Sathya Narayanan Patmanathan, Lee Fah Yap, Paul G Murray, Ian C Paterson

**Affiliations:** a Department of Oral Biology and Biomedical Sciences and Oral Cancer Research & Coordinating Centre, Faculty of Dentistry, University of MalayaKuala Lumpur, Malaysia; b School of Cancer Sciences, University of BirminghamBirmingham, UK

**Keywords:** FTY720, fingolimod, S1P, sphingosine analogue, cancer, apoptosis, cytotoxicity

## Abstract

Almost all drugs approved for use in humans possess potentially beneficial ‘off-target’ effects in addition to their principal activity. In some cases this has allowed for the relatively rapid repurposing of drugs for other indications. In this review we focus on the potential for re-purposing FTY720 (also known as fingolimod, Gilenya™), an immunomodulatory drug recently approved for the treatment of multiple sclerosis (MS). The therapeutic benefit of FTY720 in MS is largely attributed to the immunosuppressive effects that result from its modulation of sphingosine 1-phosphate receptor signalling. However, this drug has also been shown to inhibit other cancer-associated signal transduction pathways in part because of its structural similarity to sphingosine, and consequently shows efficacy as an anti-cancer agent both *in vitro* and *in vivo*. Here, we review the effects of FTY720 on signal transduction pathways and cancer-related cellular processes, and discuss its potential use as an anti-cancer drug.

- Introduction
- ‘On-target’ effects; FTY720 modulation of sphingosine-1-phosphate signalling
- ‘Off-target’ effects of FTY720
  - Sphingolipid metabolism
  - SET nuclear proto-oncogene/protein phosphatase 2A
  - Phosphatidylinositol-3 kinase (PI3K)/Akt
  - Other pathways
- Effect of FTY720 on the malignant phenotype
  - Cell death
  - Proliferation
  - Autophagy
  - Motility, invasion and metastasis
  - Epithelial to mesenchymal transition
  - Angiogenesis
  - Cancer-associated inflammation
- Second-Generation FTY720 Derivatives and Targeting Strategies
  - FTY720 derivatives that lack S1PR binding capability
  - FTY720 derivatives with enhanced SK inhibition
  - FTY720 with improved targeting
- Conclusions and future perspectives

## Introduction

**Figure 1:**
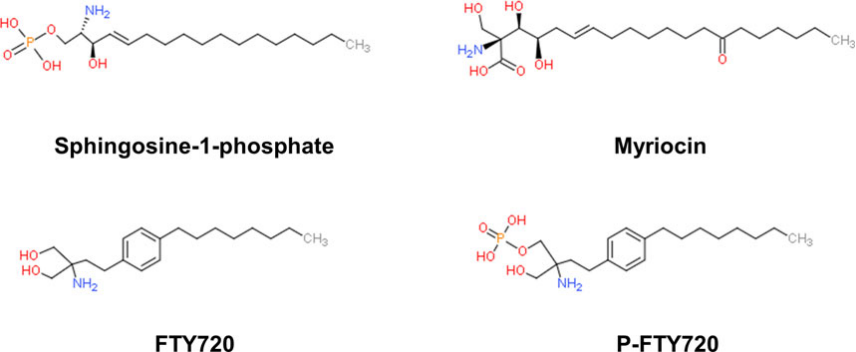
Chemical structures of sphingosine-1-phosphate, myriocin, FTY720 and phosphorylated FTY720.

**Figure 2:**
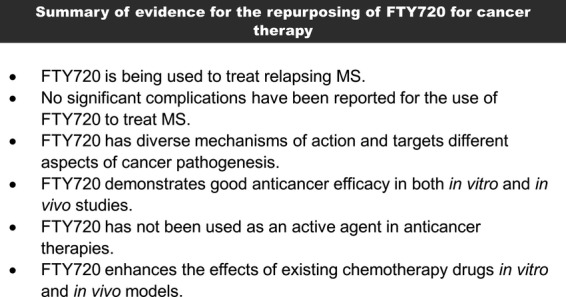
Repurposing of FTY720 for cancer therapy.

2-Amino-2-[2-(4-octylphenyl)]-1,3-propanediol hydrochloride (FTY720 or fingolimod; commercially available as Gilenya™) is an immunosuppressive drug developed by the modification of myriocin (ISP-1), a metabolite of the fungus *Isaria sinclairii* 1,2 (Fig. 1). FTY720 was found to exert its immunosuppressive effects by modulating sphingosine-1-phosphate (S1P) receptor signalling leading to sequestration of circulating lymphocytes in lymphoid tissues 3. In 2010, FTY720 was approved by the FDA as a treatment for multiple sclerosis (MS) 2. However, it has now become clear that FTY720 has a multitude of other effects on cells, many of which suggest it could be repurposed as an anti-cancer drug (Fig. 2). We now briefly review the impact of FTY720 on S1P signalling and other signal transduction pathways before considering its effects on cancer-related cellular processes.

## ‘On-target’ effects; FTY720 modulation of sphingosine-1-phosphate signalling

**Figure 3:**
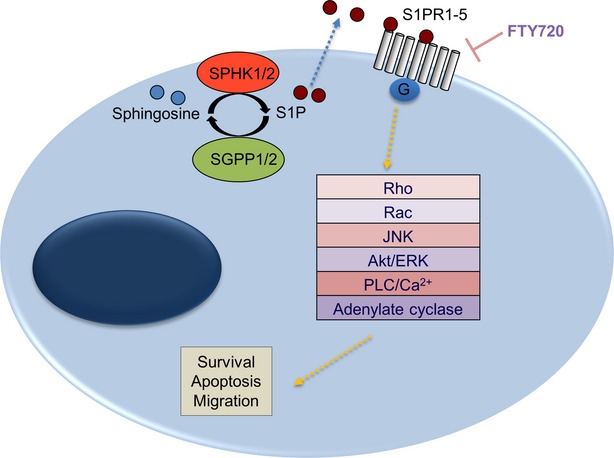
S1P signalling. S1P is generated by the sphingosine kinases, SPHK1 and SPHK2, and can be converted back to sphingosine by the S1P phosphatases, SGPP1 and SGPP2. Once secreted, S1P can act on one of at least five known S1P receptors (S1PR1-5). Activation of these receptors trigger downstream signalling, *i.e*. Rho, Rac, JNK (Jun N-terminal kinase), Akt (alpha serine/threonine-protein kinase), ERK (extracellular signal-regulated kinase), PLC (phospholipase C) and adenylate cyclase pathways, to regulate survival, apoptosis and motility of cells. FTY720 interferes with S1P signalling by binding to the S1PR1/3/4/5.

Sphingosine-1-phosphate is a small bioactive lipid which exerts its effects following binding to one or more of at least five G protein coupled receptors, known as S1PR1-5. The consequences of S1P signalling are also partly determined by the relative levels of the different receptors on the cell surface. For example, S1PR1 couples to Gi to activate Ras/ERK and PI3-kinase/Akt pathways, leading to mitogenic and pro-survival signalling and cell migration 4. In contrast, S1PR2 couples with multiple heterotrimeric G proteins, including G12/13 which exerts a potent inhibitory effect on Rac with consequent inhibition of cell migration 4. S1P functions are also regulated in part by the balance between S1P and the death-promoting sphingolipids, ceramide and sphingosine 5,6. Key regulators of this rheostat include: sphingosine kinase 1 (SPHK1) and SPHK2, which convert sphingosine to S1P; and several lipid phosphatases, including S1P phosphatase 1 and 2 (SGPP1 and SGPP2), which catalyse the conversion of S1P to sphingosine and S1P lyase, which irreversibly degrades S1P 5,6 (Fig. 3).

The classic mode of action of FTY720 is the binding of the drug to four of the S1PRs (S1PR1/3/4/5) after being phosphorylated (FTY720-P) principally by SPHK2 7. FTY720-P binds to the S1PRs at concentrations lower than 0.1 μM 8. Although FTY720 has an initial agonist activity on the receptors, it subsequently causes their internalization thereby reducing receptor levels on the cell surface 9,10. Because S1P-S1PR1 signalling is essential for T lymphocyte egress, FTY720 potently induces lymphocyte retention in peripheral lymphoid organs resulting in immunosuppression 11.

## ‘Off-target’ effects of FTY720

Apart from its classical ‘on-target’ action as a S1PR ligand, FTY720 also affects other signalling pathways when used at higher concentrations (greater than 2 μM) and we refer to these effects as ‘off-target’ actions of the drug. In the following section, we describe the main pathways that are affected by higher concentrations of FTY720.

### Sphingolipid metabolism

In part. because of a structural similarity to sphingosine, FTY720 also influences other components of the sphingolipid pathway, 12. FTY720 inhibits and reduces the expression of SPHK1 10,13–15; as a sphingosine analogue, FTY720 is a competitive inhibitor of SPHK1 and is also a non-competitive inhibitor of ATP binding to SPHK1 16. FTY720 has also been reported to inhibit and reduce the expression SPHK2 17. Further, FTY720 is a competitive inhibitor of ceramide synthase 18,19 and an inhibitor of S1P lyase 20. Therefore, as a result of its multiple effects on sphingolipid metabolism, FTY720 leads to dysregulation of ceramide, sphingosine and S1P *in vitro* and *in vivo* 17,21,22.

### SET nuclear proto-oncogene/protein phosphatase 2A

Protein phosphatase 2A (PP2A) is an enzyme with serine/threonine phosphatase activity that participates in a range of cellular mechanisms, including regulation of the cell cycle, apoptosis and cellular metabolism. Pathogenic mutations which result in decreased PP2A activity can lead to the development of colorectal and lung carcinomas and, therefore, PP2A is widely accepted as a tumour suppressor 23,24. PP2A activity is inhibited following complex formation with the SET nuclear proto-oncogene 25. FTY720 directly interferes with SET/PP2A complexes and also reduces the expression of SET, both of which ultimately lead to the reactivation of PP2A 26,27. Interestingly, although the interaction of FTY720 with SET/PP2A is independent of S1PRs, FTY720-P can also suppress PP2A activity *via* S1PR1 28,29.

### Phosphatidylinositol-3 kinase/Akt

The phosphatidylinositol-3 kinase (PI3K)/Akt pathway participates in the regulation of cell metabolism, proliferation and survival, often *via* extensive crosstalk with other signalling pathways (including S1P and PP2A signalling) 30. Upon activation, PI3K phosphorylates its substrate PIP2 to generate PIP3, which then activates Akt (v-akt murine thymoma viral oncogene homologue; protein kinase B). P13K signalling is disrupted in cancer following mutation of the PI3K gene itself or of other molecules that regulate its activity. One such molecule is phosphatase and tensin homologue deleted on chromosome 10 (PTEN), a tumour suppressor that inhibits Akt activation 30. FTY720 mediates many of its anti-cancer effects through inactivation of the PI3K/Akt pathway 31–34 mediated *via* a variety of mechanisms which include the inhibition of PI3K 35, increased PTEN expression 36, activation of PP2A 37–39 and SPHK1 inhibition 14,33,34. It is important to note that as the PI3K/Akt pathway can also be activated by S1P, it is likely that the inhibition of this pathway by FTY720 could occur *via* both S1P-dependent and -independent mechanisms.

### Other pathways

14-3-3 proteins are a family of seven protein isoforms whose activities depend on the phosphorylation of serine/threonine residues. Once activated, these molecules bind with a diverse group of proteins that participate in signal transduction, which allows 14-3-3 proteins to regulate a wide range of regulatory processes, such as cell cycle 40 and apoptosis 41. Similar to sphingosine, FTY720 directly modulates 14-3-3 proteins to facilitate their phosphorylation by protein kinase A (PKA) and possibly protein kinase C δ 42, thereby influencing a vast array of cellular activities.

Reactive oxygen species (ROS) are generated as by-products of normal metabolism and are important regulators of cell signalling 43. FTY720 has been shown to increase the permeabilization of lysosomal membranes and augment ROS release into the cytoplasm 21,44. Other studies showed that FTY720 can increase ROS production 45–47 and this was found to be essential for the down-regulation of the anti-apoptotic protein, Mcl-1 in natural killer (NK) leukaemia cells 21, as well as for the activation of pro-apoptotic PKCδ in hepatocellular carcinoma 34.

## Effect of FTY720 on the malignant phenotype

### Cell death

FTY720 is cytotoxic and efficiently reduces the viability of cancer cell lines *in vitro* (IC50s in the range 5-20 μM), such as those from ovarian 13,48, colorectal 31,49,50, breast 45,50–52 prostate 22,53 and blood cancers 28,38,39,46,54–56, amongst others 57. In some *in vitro* studies, FTY720 shows selective killing of neoplastic cells while having minimal effects on normal cells 35,51,52,56,58–61; effects which can be recapitulated in cancer mouse models in which FTY720 (used at 2.5–10 mg/kg) was shown to reduce tumour burden and prolong survival without causing significant damage to non-diseased organs 28,39,52,60–64.

In the majority of studies, the cytotoxicity of FTY720 was shown to be because of its ability to induce apoptosis. Cells treated with FTY720 frequently show caspase-3, -8 and -9 activation, implicating FTY720 in both extrinsic and intrinsic apoptotic pathways 31,33,55,65–67. FTY720 differentially modulates the Bcl-2 family of regulatory proteins to facilitate apoptosis. For example, FTY720 down-regulates the anti-apoptotic proteins Bcl-2, Bcl-xL and Mcl-1 60,65,68 and up-regulates Bax and Bad which are pro-apoptotic 55,60,65. FTY720 also down-regulates the apoptotic inhibitor, survivin 65,68 and up-regulates the pro-apoptotic BH3-only proteins, Bim and Bid 33,36,69.

Protein phosphatase 2A activation appears to be essential in mediating the apoptosis induced by FTY720 in several haematological cancers, because inhibition of PP2A activity by okadaic acid rescued cell death induced by FTY720 32,38,39. ROS generation also contributes to apoptosis as FTY720 induced apoptosis can be partially rescued with a ROS scavenger 34,45,68. Furthermore, 14-3-3 phosphorylation was shown to be important in mediating FTY720-induced apoptosis, because cell death was attenuated following transfection with a non-phosphorylatable 14-3-3zeta mutant 42. FTY720 interactions with the S1PRs appear not to be involved in the apoptotic response because FTY720-P (which binds to S1PRs) did not kill a variety of cancer cell types that were sensitive to FTY720 17,50,59,69. Moreover, pre-treatment of B-cell chronic lymphocytic leukemia (B-CLL) cells with S1P failed to alter the cytotoxic effects of FTY720 38. Nevertheless, the sphingolipid pathway may play a role in mediating the cytotoxic effects of FTY720 in some circumstances *via* the inhibition of SPHK1 by FTY720. For example, overexpression of SPHK1 rescued prostate cancer cells from FTY720-induced cell death, however, this effect was not observed in cells with silenced S1PRs 22. These results suggest that SPHK1 inhibition, but not the interaction with S1PRs, may be important in mediating the cytotoxic effects of FTY720.

Necrotic cell death induced by FTY720 has also been observed. FTY720-treated ovarian and melanoma cells showed no evidence of caspase activation and the cells were not able to bind to Annexin V 48,62. Similarly, death induced in the cell lines of neuroblastoma, acute lymphoblastic leukaemia cells, mantle cell lymphoma, other and B cell malignancies were also caspase-independent, although necrosis was not proven 17,38,46,47. Further, in an interleukin (IL)-3 dependent murine haematopoietic cell line, FL5.12, in which apoptosis was disabled by overexpressing Bcl-2, FTY720 down-regulated nutrient transporter proteins which resulted in starvation-induced necrosis 59. ROS production has also been identified as an important mechanism for FTY720-induced necrosis 46,47,62 FTY720 can also induce receptor interacting protein kinase 1-dependent necroptosis following the activation of PP2A 29. Together, these findings demonstrate the ability of FTY720 to kill cancer cells by different mechanisms in a variety of cellular settings.

Some cancer cells that are resistant to conventional chemotherapy appear to be sensitive to FTY720. For example, FTY720 can kill imatinib-resistant gastrointestinal stromal tumour 70 and myeloid cells harbouring c-KIT mutations. Similarly, FTY720 was cytotoxic towards leukaemic cells that demonstrate resistance to tyrosine kinase inhibitors 33,39. FTY720 could also kill ovarian cancer cells independent of their sensitivity towards cisplatin, paclitaxel or other chemotherapy 13,48. Studies using FTY720 in combination with a variety of conventional chemotherapy agents have demonstrated additive or synergistic effects (Table 1). FTY720 has also shown a convincing ability to sensitize cancer cells to radiation; FTY720 reduced the activation of Akt and down-regulated survivin, both of which were induced by radiation and were implicated in the radio-resistance of a breast cancer cell line 71. In addition, FTY720 increased the radio-sensitivity of prostate cancer cells overexpressing miR-95, a microRNA associated with resistance to radiation) 72. Similarly, the combination of FTY720 and radiation showed enhanced SK1 inhibition and tumour suppression in a mouse xenograft model of prostate cancer 22.

**Table 1:** Combinatorial effects of FTY720 and chemotherapy drugs

Chemotherapy	Type of study	Type of malignancy	Proposed mechanism(s)	References
5-Fluorouracil, SN-38, and oxaliplatin	*In vitro*	Colorectal	SET/PP2A, PI3K/Akt	31
Cisplatin	*In vivo*	Lung	SET/PP2A, NDRG1	85
Doxorubicin and etoposide	*In vitro*	Colon	Inhibition of P-glycoprotein (P-gp) and multidrug resistance protein 1 (MRP1)	49
Doxorubicin	*In vivo*	Leukaemia	SET/PP2A	110
Topotecan	*In vitro* and *in vivo*	Neuroblastoma	SK2, PI3K/Akt	17
Cetuximab	*In vitro* and *in vivo*	Colorectal	SK1	14
Temozolomide	*In vivo*	Brain tumour stem cell	–	69
Milatuzumab	*In vitro* and *in vivo*	Mantle cell lymphoma	Lysosomal membrane permeabilization	44
Nanoliposomal C6-ceramide	*In vitro*	NK-cell leukaemia	ROS, sphingolipid pathway	21
Sunitinib malate	*In vivo*	Breast	S1PR1/3 antagonizm	88
Cisplatin	*In vitro*	Gastric	PTEN/PI3K/Akt	36
Rapamycin	*In vitro*	Pancreas	–	111

### Proliferation

At cytotoxic concentrations, FTY720 has also been shown to induce G1 arrest by modulating key cell cycle regulators. For example, FTY720 down-regulates cyclin D1, cyclin E 35,47,65 and cyclin-dependant kinase (CDK)2/4, and up-regulates the CDK inhibitors, p16, p21, p27, 35,36,65,67. In addition, the retinoblastoma protein (pRb) was found to be in its inactive dephosphorylated state in FTY720 treated cells 73. Both PP2A 29,32 and PTEN/PI3K/Akt 35,36 signalling pathways have been shown to mediate FTY720-induced growth suppression.

### Autophagy

Autophagy is a physiological process in which damaged organelles form an autophagosome which is subsequently digested by lysosomal enzymes. The resulting metabolites are either recycled or used as a short-term energy supply in times of cellular stress. Autophagy plays an ambiguous role in cancer progression, as it can induce prolonged survival of cancer cells by conserving energy or lead to cell death 74. FTY720 can increase the accumulation of autophagosomes in many malignancies either by inducing autophagosome formation 46,48,59,68 or by blocking the fusion of autophagosomes and lysosomes (autophagic flux) 44. FTY720-induced autophagy was found to be protective against the cytotoxic nature of FTY720, which was demonstrated by the ability of 3-methyladenine, an autophagy inhibitor, to enhance the cell death induced by FTY720 46,48,62. In addition, autophagy-deficient murine embryonic fibroblasts were more sensitive to FTY720-induced cytotoxicity 59. Interestingly, FTY720-P was also found to induce autophagy 46, implying the involvement of S1P and the S1PRs. In acute lymphoblastic leukaemia cells, FTY720 was shown to mediate autophagy by down-regulating Mcl-1, which inhibits Beclin-1 46, an important inducer of autophagy 74. Similarly, Beclin-1 was up-regulated by FTY720 in ovarian cancer cells 48. By contrast, FTY720-induced autophagy promoted apoptosis in multiple myeloma cells in which both autophagy and apoptosis were mediated through ROS generation, an effect that was attributed to the degradation of anti-apoptotic protein, Mcl-1 and survivin 68.

### Motility, invasion and metastasis

At concentrations below those that cause cytotoxicity, FTY720 treatment decreased the migration and invasive ability of glioblastoma and prostate cancer cells in *in vitro* assays 53,75,76. The anti-migratory and/or anti-invasive effects of FTY720 have also been reported in other cancer cell lines, such as those from ovarian cancer 13, hepatocellular carcinoma 77–79, pancreatic cancer 80 and cholangiocarcinoma 65. These results are supported by observations that FTY720 induced cytoskeletal disorganisation in prostate cancer cells 53 and also decreased and deformed microfilaments, filopodia and microvilli on the cell surface of murine breast cancer cell lines 52. In addition, FTY720 has been shown to suppress lymph node and organ metastasis in many *in vivo* cancer models 45,52,65,69,79,81, indicating that FTY720 might be effective in managing late stage disease.

The concentration of FTY720 needed for the drug to inhibit tumour cell migration/invasion is lower than that required to induce cytotoxicity in both *in vitro* (2 μM or less) 13,53,61,75,78 and *in vivo* studies (2 mg/kg) 52. This suggests, therefore, that the SPHK1/S1P/S1PR signalling pathway is important in mediating the effects of FTY720 on migration/invasion and metastasis. FTY720-P inhibited S1P-induced migration of classical Hodgkin lymphoma cells by modulating S1PR1 82 and SK1 inhibition with FTY720 reduced the migration of ovarian cancer cells 13. FTY720 affects a number of pathways that are known to be downstream of the S1PRs, such as the Rho family of small GTPases, which are important regulators of cell mobility 83. FTY720 down-regulated the active form of RhoA in pancreatic cancer cells 53, reduced levels of active Rac in hepatocellular carcinoma 78,79 as well as decreasing levels of ROBO1 and ROCK1 (targets of RhoA) in glioblastoma cells. FTY720 also decreased the expression of metalloproteinases (MMP-2 and MMP-9) and increased tissue inhibitors of metalloproteinases (TIMP-1 and TIMP-2) 13,75. The PI3K/Akt pathway has been implicated in FTY720-induced motility 75,78, although this could also be the downstream of S1PRs.

### Epithelial to mesenchymal transition

Epithelial to mesenchymal transition (EMT) is a process by which epithelial cells undergo molecular and morphologic changes to resemble the mesenchymal phenotype, which leads to the acquisition of migratory and invasive capacity, evasion of apoptosis and senescence, as well as ability to resist chemotherapy 84. The growth of xenografts derived from cisplatin resistant lung cancer cells showing features of EMT was suppressed by FTY720, both alone or in combination with cisplatin 85. These effects were attributed to the ability of FTY720 to modulate the PP2A/SET interaction together with a concomitant increase in E-cadherin and the Snail transcription factor, as well as a decrease in vimentin expression 85. Similar observations were made in cholangiocarcinoma 65 and glioblastoma 75, where FTY720-treated cells showed higher expression of E-cadherin and reduced expression of N-cadherin, vimentin and Twist1 65,75. In androgen-independent prostate cancer cells, Runx2 modulates EMT by switching of E-cadherin to N-cadherin and FTY720 down-regulated Runx2 thereby reversing the cadherin switch 76.

### Angiogenesis

Angiogenesis is the process by which new blood vessels are formed to sustain nutrient and oxygen requirements of actively proliferating cells and is important for the sustained growth of most tumours 86. FTY720 has been reported to inhibit angiogenesis in several xenograft cancer models 61,65,79,81. Similarly, FTY720 attenuated both S1P- and VEGF-driven angiogenesis in an agar chamber model *in vivo* 81 and a Matrigel plug *in vivo* assay for Lewis lung carcinoma 87. Furthermore, FTY720 normalized the vasculature within mammary tumours in rats 88 and abrogated increased vascular permeability 61,81, both of which can promote the cytotoxic effects of chemotherapy and radiotherapy 89,90. In addition, low doses of FTY720 did not kill B16/BL6 melanoma cells *in vitro* but reduced the growth of these cells and inhibited neovascularization *in vivo*, suggesting the indirect killing of tumour cells by reducing tumour vascularity 81.

FTY720 has been shown to inhibit angiogenesis by a number of mechanisms. For example, FTY720 was found to reduce the migration of human umbilical vein endothelial cells (HUVEC) 61,63,81,87 and to block the recruitment of vascular smooth muscle cells (VSMC) by S1P, endothelial cells or tumour cells 88. S1PR antagonizm by FTY720 is important in mediating these anti-angiogenic effects as they are induced at low doses 61,81,87 and by FTY720-P 81. In support of these observations, FTY720 reversed the effect of S1P on VSMC/HUVEC migration and the formation of blood vessels by a mechanism involving S1PR1/3 81,88,91. In addition, FTY720 down-regulates VEGF, an important angiogenic inducer 63,92 as well as reduces the expression of chemokines, i.e. CXCL10, CXCR3 and CXCR4 92.

### Cancer-associated inflammation

It is now recognized that inflammation can promote tumourigenesis 93. FTY720 suppressed azoxymethane-induced colonic inflammation in mice and suppressed the subsequent development of tumours by down-regulating SPHK1 and S1PR1, which is important for persistent NF-κB and STAT3 activation, as well as IL-6 production in this model 10. In addition, FTY720 has been reported to down-regulate the pro-inflammatory mediators CXCL10, VEGF, CXCR4 and CXCR3 and reduce hepatic ischaemia-reperfusion injury, which otherwise contributes to metastasis in rats with hepatic tumours 92.

## Second-generation FTY720 derivatives and targeting strategies

### FTY720 derivatives that lack S1PR binding capability

As many of the anti-cancer effects of FTY720 are independent of S1PRs and there are possible side effects associated with antagonizing S1P signalling, a non-immunosuppressive FTY720 analogue, OSU-2S was developed that cannot be phosphorylated by SPHK2 and does not induce S1PR1 internalization 98. Compared to FTY720, OSU-2S demonstrated more cytotoxicity and selectivity (in relation to normal liver cells) in hepatocellular carcinoma, both *in vitro* and *in vivo*, 98. OSU-2S was also shown to induce cytotoxicity in CLL 99. Two other FTY720 derivatives, (S)-FTY720-OMe, (S)-FTY720-regioisomer, were found to reduce survival of chronic myeloid leukemia (CML) haematopoietic stem cells (HSC) but not normal HSCs 58 and caused PP2A activation without stimulating S1PR1 internalization and B cell lymphopenia 58. Both OSU-2S and (S)-FTY720-regioisomer were also found to be more potent than FTY720 in reducing the clonogenic survival of Jak2-driven haematological malignancies 28.

Another FTY720 analogue devoid of the ability to be phosphorylated is AAL-149 59. This drug demonstrated the same potency and mechanism of selective cytoxicity as FTY720 in patient-derived leukaemic cells 59. In 2013, Fransson and colleagues designed novel stereochemically constrained analogues of FTY720 and showed that one of these analogues had an enhanced anti-leukaemic activity compared to FTY720 100. However, the same enhanced potency was not observed for prostate cancer cells 100.

### FTY720 derivatives with enhanced sphingosine kinase inhibition

Efforts have also been made to chemically modify FTY720 to improve its efficacy as a SPHK inhibitor. This has resulted in the generation of two compounds; (S)-FTY720 vinylphosphonate and (R)-FTY720 methyl ether (ROME). (S)-FTY720 vinylphosphonate inhibits and reduces the expression of SPHK1 15,16,101,102, resulting in apoptosis of prostate cancer cells 101 and human pulmonary smooth muscle cells 15. In addition to inhibiting SPHK1, (S)-FTY720 vinylphosphonate abrogated the S1P-stimulated rearrangement of actin in breast cancer cells 103. ROME, on the other hand, is a derivative which selectively inhibits and down-regulates SPHK2 104,105 in turn inhibiting DNA synthesis and preventing S1P-mediated rearrangement of actin in MCF-7 cells 105. However, ROME did not induce apoptosis in androgen-sensitive LNCaP prostate cancer cells 106.

### FTY720 with improved targeting

To reduce unwanted toxicity, recent studies have also examined the feasibility of improved targeting of FTY720. Liposomal formulation of FTY720 improved the stability of FTY720 in aqueous buffer without affecting the cytotoxicity of CLL cells 107. When this formulation was coupled to an antibody (*i.e*. CD19, CD20 and CD37), a superior specificity against CLL cells was observed 107. Similarly, liposomal-antibody packaging of OSU-2S allowed this drug to selectively target CLL cells, sparing normal B cells 99. Dual antibody immunoliposomes have been developed as vehicles for targeted delivery 108, which resulted in enhanced delivery of FTY720 and increased apoptosis in CLL cells compared to the single antibody liposomal targeting 108.

## Conclusions and future perspectives

**Figure 4:**
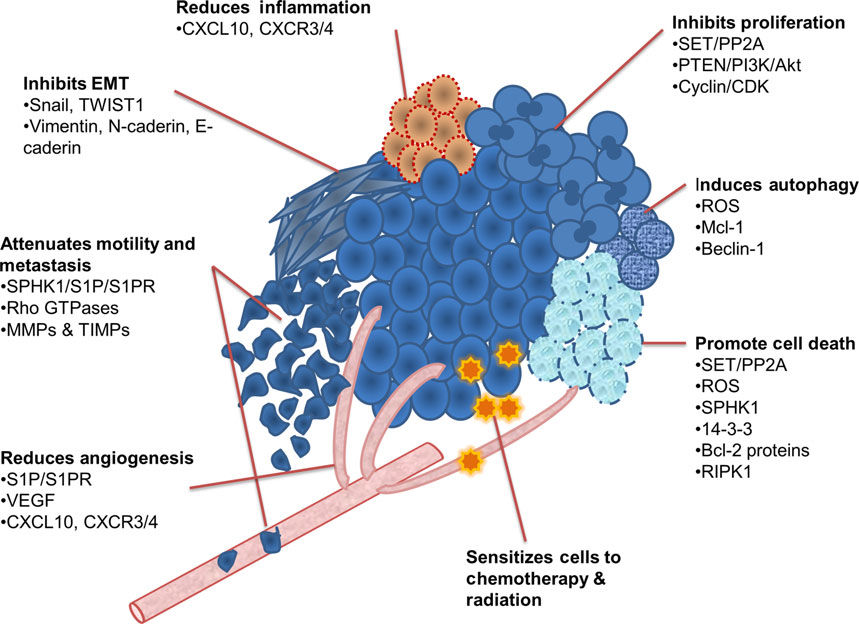
Effects of FTY720 on cancer cells.

The ability of FTY720 to target multiple signalling pathways which control cell proliferation, death, motility, angiogenesis and inflammation (Fig. 4), suggests that this drug is not only likely to be useful against a wide range of tumours containing different molecular abnormalities, but also that it could reduce the likelihood of resistance resulting from the activation of other compensatory pathways 94; using a single drug that targets multiple pathways would seem to be an attractive alternative to the use of combinations of drugs with narrower specificity to reduce the likelihood of developing resistant disease 94. The toxicity profile of FTY720 is well described in MS patients and includes immunosuppression, bradycardia and increased risk of melanoma 95–97. However, it is difficult to predict the toxicity associated with the use of FTY720 in cancer patients, because dose and duration of treatment may be different; there is already evidence from *in vitro* studies that the dose required to achieve an anticancer effect is higher than that necessary to antagonize S1PR signalling. The long-term adverse effects of FTY720 treatment are still to be fully determined, but will obviously be important considerations for the potential future use of this drug in cancer patients.

By targeting a range of processes implicated in tumourigenesis, FTY720 is a promising anticancer agent across a broad range of malignancies that has the potential and meets a number of accepted criteria for drug repurposing 109 (Fig. 2). The second-generation derivatives of FTY720 have higher efficacy, lower toxicity and better selectivity (Table 2). However, as the precise effects of FTY720 on molecular signalling pathways and clinical phenotypes appear to be cell-type dependent, further studies are required to fully evaluate the utility of FTY720 and its derivatives in different cancer settings.

**Table 2:**
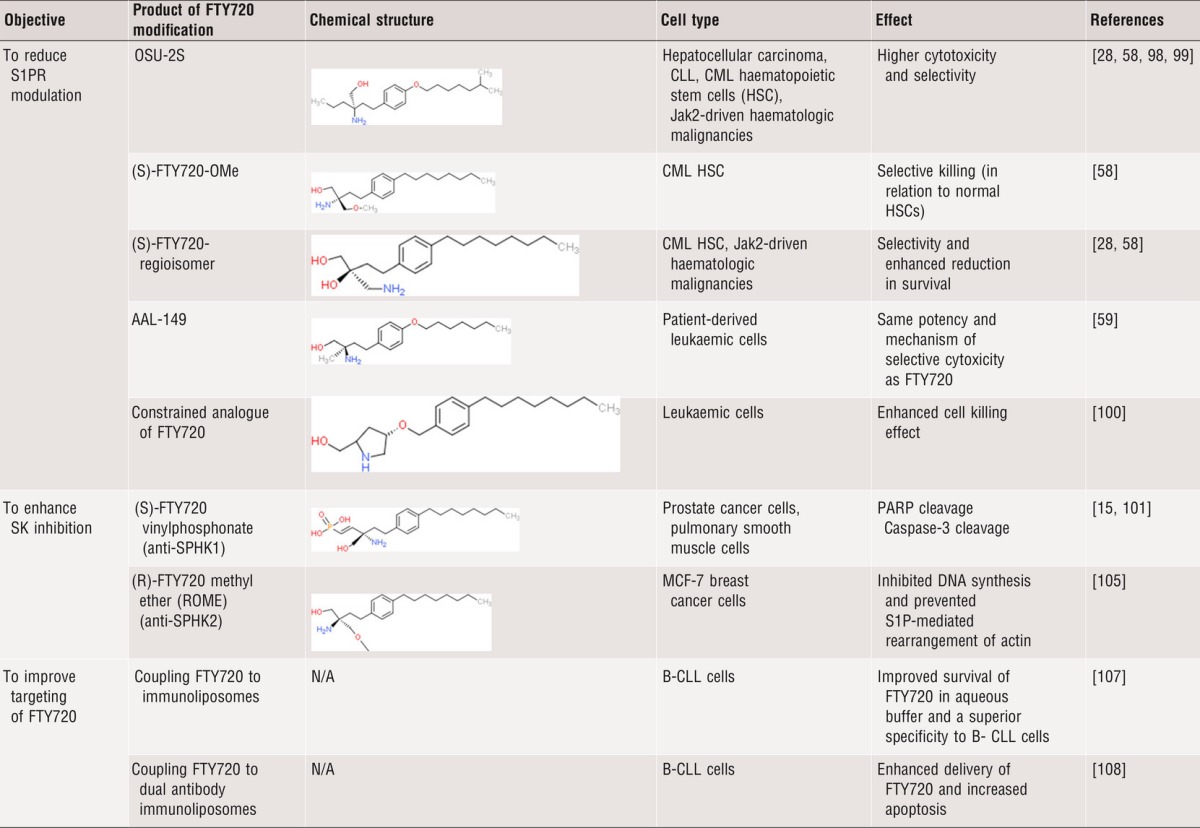
The effects of second-generation derivatives of FTY720 on cancer

## References

[b1] Fujita T, Inoue K, Yamamoto S (1994). Fungal metabolites. Part 11. A potent immunosuppressive activity found in *Isaria sinclairii* metabolite. J Antibiot.

[b2] Strader CR, Pearce CJ, Oberlies NH (2011). Fingolimod (FTY720): a recently approved multiple sclerosis drug based on a fungal secondary metabolite. J Nat Prod.

[b3] Chun J, Hartung HP (2010). Mechanism of action of oral fingolimod (FTY720) in multiple sclerosis. Clin Neuropharmacol.

[b4] Adada M, Canals D, Hannun YA (2013). Sphingosine-1-phosphate receptor 2. FEBS J.

[b5] Ponnusamy S, Meyers-Needham M, Senkal CE (2010). Sphingolipids and cancer: ceramide and sphingosine-1-phosphate in the regulation of cell death and drug resistance. Future Oncol.

[b6] Pyne S, Pyne NJ (2013). New perspectives on the role of sphingosine 1-phosphate in cancer. Handb Exp Pharmacol.

[b7] Pitman MR, Woodcock JM, Lopez AF (2012). Molecular targets of FTY720 (fingolimod). Curr Mol Med.

[b8] Brinkmann V, Davis MD, Heise CE (2002). The immune modulator FTY720 targets sphingosine 1-phosphate receptors. J Biol Chem.

[b9] Mehling M, Johnson TA, Antel J (2011). Clinical immunology of the sphingosine 1-phosphate receptor modulator fingolimod (FTY720) in multiple sclerosis. Neurology.

[b10] Liang J, Nagahashi M, Kim EY (2013). Sphingosine-1-phosphate links persistent STAT3 activation, chronic intestinal inflammation, and development of colitis-associated cancer. Cancer Cell.

[b11] Chiba K, Matsuyuki H, Maeda Y (2006). Role of sphingosine 1-phosphate receptor type 1 in lymphocyte egress from secondary lymphoid tissues and thymus. Cell Mol Immunol.

[b12] Brinkmann V, Billich A, Baumruker T (2010). Fingolimod (FTY720): discovery and development of an oral drug to treat multiple sclerosis. Nat Rev Drug Discov.

[b13] Lee JW, Ryu JY, Yoon G (2014). Sphingosine kinase 1 as a potential therapeutic target in epithelial ovarian cancer. Int J Cancer.

[b14] Rosa R, Marciano R, Malapelle U (2013). Sphingosine kinase 1 overexpression contributes to cetuximab resistance in human colorectal cancer models. Clin Cancer Res.

[b15] Tonelli F, Lim KG, Loveridge C (2010). FTY720 and (S)-FTY720 vinylphosphonate inhibit sphingosine kinase 1 and promote its proteasomal degradation in human pulmonary artery smooth muscle, breast cancer and androgen-independent prostate cancer cells. Cell Signal.

[b16] Lim KG, Tonelli F, Berdyshev E (2012). Inhibition kinetics and regulation of sphingosine kinase 1 expression in prostate cancer cells: functional differences between sphingosine kinase 1a and 1b. Int J Biochem Cell Biol.

[b17] Li MH, Hla T, Ferrer F (2013). FTY720 inhibits tumor growth and enhances the tumor-suppressive effect of topotecan in neuroblastoma by interfering with the sphingolipid signaling pathway. Pediatr Blood Cancer.

[b18] Kim HJ, Qiao Q, Toop HD (2012). A fluorescent assay for ceramide synthase activity. J Lipid Res.

[b19] Lahiri S, Park H, Laviad EL (2009). Ceramide synthesis is modulated by the sphingosine analog FTY720 *via* a mixture of uncompetitive and noncompetitive inhibition in an Acyl-CoA chain length-dependent manner. J Biol Chem.

[b20] Bandhuvula P, Tam YY, Oskouian B (2005). The immune modulator FTY720 inhibits sphingosine-1-phosphate lyase activity. J Biol Chem.

[b21] Liao A, Broeg K, Fox T (2011). Therapeutic efficacy of FTY720 in a rat model of NK-cell leukemia. Blood.

[b22] Pchejetski D, Bohler T, Brizuela L (2010). FTY720 (fingolimod) sensitizes prostate cancer cells to radiotherapy by inhibition of sphingosine kinase-1. Cancer Res.

[b23] Chen W, Wang Z, Jiang C (2013). PP2A-mediated anticancer therapy. Gastroenterol Res Pract.

[b24] Walter G, Ruediger R (2012). Mouse model for probing tumor suppressor activity of protein phosphatase 2A in diverse signaling pathways. Cell Cycle.

[b25] Li M, Makkinje A, Damuni Z (1996). The myeloid leukemia-associated protein SET is a potent inhibitor of protein phosphatase 2A. J Biol Chem.

[b26] Pippa R, Dominguez A, Christensen DJ (2014). Effect of FTY720 on the SET-PP2A complex in acute myeloid leukemia; SET binding drugs have antagonistic activity. Leukemia.

[b27] Yang Y, Huang Q, Lu Y (2012). Reactivating PP2A by FTY720 as a novel therapy for AML with C-KIT tyrosine kinase domain mutation. J Cell Biochem.

[b28] Oaks JJ, Santhanam R, Walker CJ (2013). Antagonistic activities of the immunomodulator and PP2A-activating drug FTY720 (Fingolimod, Gilenya) in Jak2-driven hematologic malignancies. Blood.

[b29] Saddoughi SA, Gencer S, Peterson YK (2013). Sphingosine analogue drug FTY720 targets I2PP2A/SET and mediates lung tumour suppression *via* activation of PP2A-RIPK1-dependent necroptosis. EMBO Mol Med.

[b30] Jiang BH, Liu LZ (2009). PI3K/PTEN signaling in angiogenesis and tumorigenesis. Adv Cancer Res.

[b31] Cristobal I, Manso R, Rincon R (2014). PP2A inhibition is a common event in colorectal cancer and its restoration using FTY720 shows promising therapeutic potential. Mol Cancer Ther.

[b32] Roberts KG, Smith AM, McDougall F (2010). Essential requirement for PP2A inhibition by the oncogenic receptor c-KIT suggests PP2A reactivation as a strategy to treat c-KIT^+^ cancers. Cancer Res.

[b33] Kiyota M, Kuroda J, Yamamoto-Sugitani M (2013). FTY720 induces apoptosis of chronic myelogenous leukemia cells *via* dual activation of BIM and BID and overcomes various types of resistance to tyrosine kinase inhibitors. Apoptosis.

[b34] Hung JH, Lu YS, Wang YC (2008). FTY720 induces apoptosis in hepatocellular carcinoma cells through activation of protein kinase C delta signaling. Cancer Res.

[b35] Lee TK, Man K, Ho JW (2004). FTY720 induces apoptosis of human hepatoma cell lines through PI3-K-mediated Akt dephosphorylation. Carcinogenesis.

[b36] Zheng T, Meng X, Wang J (2010). PTEN- and p53-mediated apoptosis and cell cycle arrest by FTY720 in gastric cancer cells and nude mice. J Cell Biochem.

[b37] Cristobal I, Garcia-Orti L, Cirauqui C (2011). PP2A impaired activity is a common event in acute myeloid leukemia and its activation by forskolin has a potent anti-leukemic effect. Leukemia.

[b38] Liu Q, Zhao X, Frissora F (2008). FTY720 demonstrates promising preclinical activity for chronic lymphocytic leukemia and lymphoblastic leukemia/lymphoma. Blood.

[b39] Neviani P, Santhanam R, Oaks JJ (2007). FTY720, a new alternative for treating blast crisis chronic myelogenous leukemia and Philadelphia chromosome-positive acute lymphocytic leukemia. J Clin Invest.

[b40] Dar A, Wu D, Lee N (2014). 14-3-3 proteins play a role in the cell cycle by shielding cdt2 from ubiquitin-mediated degradation. Mol Cell Biol.

[b41] Masters SC, Fu H (2001). 14-3-3 proteins mediate an essential anti-apoptotic signal. J Biol Chem.

[b42] Woodcock JM, Ma Y, Coolen C (2010). Sphingosine and FTY720 directly bind pro-survival 14-3-3 proteins to regulate their function. Cell Signal.

[b43] Nogueira V, Hay N (2013). Molecular pathways: reactive oxygen species homeostasis in cancer cells and implications for cancer therapy. Clin Cancer Res.

[b44] Alinari L, Mahoney E, Patton J (2011). FTY720 increases CD74 expression and sensitizes mantle cell lymphoma cells to milatuzumab-mediated cell death. Blood.

[b45] Pereira FV, Arruda DC, Figueiredo CR (2013). FTY720 induces apoptosis in B16F10-NEX2 murine melanoma cells, limits metastatic development *in vivo*, and modulates the immune system. Clinics.

[b46] Wallington-Beddoe CT, Hewson J, Bradstock KF (2011). FTY720 produces caspase-independent cell death of acute lymphoblastic leukemia cells. Autophagy.

[b47] Liu Q, Alinari L, Chen CS (2010). FTY720 shows promising *in vitro* and *in vivo* preclinical activity by downmodulating Cyclin D1 and phospho-Akt in mantle cell lymphoma. Clin Cancer Res.

[b48] Zhang N, Qi Y, Wadham C (2010). FTY720 induces necrotic cell death and autophagy in ovarian cancer cells: a protective role of autophagy. Autophagy.

[b49] Xing Y, Wang ZH, Ma DH (2014). FTY720 enhances chemosensitivity of colon cancer cells to doxorubicin and etoposide *via* the modulation of P-glycoprotein and multidrug resistance protein 1. J Dig Dis.

[b50] Nagaoka Y, Otsuki K, Fujita T (2008). Effects of phosphorylation of immunomodulatory agent FTY720 (fingolimod) on antiproliferative activity against breast and colon cancer cells. Biol Pharm Bull.

[b51] Azuma H, Horie S, Muto S (2003). Selective cancer cell apoptosis induced by FTY720; evidence for a Bcl-dependent pathway and impairment in ERK activity. Anticancer Res.

[b52] Azuma H, Takahara S, Ichimaru N (2002). Marked prevention of tumor growth and metastasis by a novel immunosuppressive agent, FTY720, in mouse breast cancer models. Cancer Res.

[b53] Zhou C, Ling MT, Kin-Wah Lee T (2006). FTY720, a fungus metabolite, inhibits invasion ability of androgen-independent prostate cancer cells through inactivation of RhoA-GTPase. Cancer Lett.

[b54] Liu Y, Deng J, Wang L (2012). S1PR1 is an effective target to block STAT3 signaling in activated B cell-like diffuse large B-cell lymphoma. Blood.

[b55] Yasui H, Hideshima T, Raje N (2005). FTY720 induces apoptosis in multiple myeloma cells and overcomes drug resistance. Cancer Res.

[b56] Shah MV, Zhang R, Irby R (2008). Molecular profiling of LGL leukemia reveals role of sphingolipid signaling in survival of cytotoxic lymphocytes. Blood.

[b57] Zhang L, Wang HD, Ji XJ (2013). FTY720 for cancer therapy. Oncol Rep.

[b58] Neviani P, Harb JG, Oaks JJ (2013). PP2A-activating drugs selectively eradicate TKI-resistant chronic myeloid leukemic stem cells. J Clin Invest.

[b59] Romero Rosales K, Singh G, Wu K (2011). Sphingolipid-based drugs selectively kill cancer cells by down-regulating nutrient transporter proteins. Biochem J.

[b60] Ubai T, Azuma H, Kotake Y (2007). FTY720 induced Bcl-associated and Fas-independent apoptosis in human renal cancer cells *in vitro* and significantly reduced *in vivo* tumor growth in mouse xenograft. Anticancer Res.

[b61] Ho JW, Man K, Sun CK (2005). Effects of a novel immunomodulating agent, FTY720, on tumor growth and angiogenesis in hepatocellular carcinoma. Mol Cancer Ther.

[b62] Tay KH, Liu X, Chi M (2014). Involvement of vacuolar H -ATPase in killing of human melanoma cells by the sphingosine kinase analogue FTY720. Pigment Cell Melanoma Res.

[b63] Chua CW, Lee DT, Ling MT (2005). FTY720, a fungus metabolite, inhibits *in vivo* growth of androgen-independent prostate cancer. Int J Cancer.

[b64] Azuma H, Takahara S, Horie S (2003). Induction of apoptosis in human bladder cancer cells *in vitro* and *in vivo* caused by FTY720 treatment. J Urol.

[b65] Lu Z, Wang J, Zheng T (2014). FTY720 inhibits proliferation and epithelial-mesenchymal transition in cholangiocarcinoma by inactivating STAT3 signaling. BMC Cancer.

[b66] Lee YS, Nakajima H, Tsuruga M (2003). Elimination of cell-cycle regulators during caspase-3-dependent apoptosis caused by an immunosuppressant, FTY720. Biosci Biotechnol Biochem.

[b67] Permpongkosol S, Wang JD, Takahara S (2002). Anticarcinogenic effect of FTY720 in human prostate carcinoma DU145 cells: modulation of mitogenic signaling, FAK, cell-cycle entry and apoptosis. Int J Cancer.

[b68] Liao A, Hu R, Zhao Q (2012). Autophagy induced by FTY720 promotes apoptosis in U266 cells. Eur J Pharm Sci.

[b69] Estrada-Bernal A, Palanichamy K, Ray Chaudhury A (2012). Induction of brain tumor stem cell apoptosis by FTY720: a potential therapeutic agent for glioblastoma. Neuro Oncol.

[b70] Dumont AG, Reynoso DG, Trent JC (2011). Essential requirement for PP2A inhibition by the oncogenic receptor c-KIT suggests PP2A reactivation as a strategy to treat c-KIT^+^ cancers – Letter. Cancer Res.

[b71] Marvaso G, Barone A, Amodio N (2014). Sphingosine analog fingolimod (FTY720) increases radiation sensitivity of human breast cancer cells *in vitro*. Cancer Biol Ther.

[b72] Huang X, Taeb S, Jahangiri S (2013). miRNA-95 mediates radioresistance in tumors by targeting the sphingolipid phosphatase SGPP1. Cancer Res.

[b73] Nagahara Y, Matsuoka Y, Saito K (2001). Coordinate involvement of cell cycle arrest and apoptosis strengthen the effect of FTY720. Jpn J Cancer Res.

[b74] Kenific CM, Debnath J (2015). Cellular and metabolic functions for autophagy in cancer cells. Trends Cell Biol.

[b75] Zhang L, Wang H, Zhu J (2014). FTY720 reduces migration and invasion of human glioblastoma cell lines *via* inhibiting the PI3K/AKT/mTOR/p70S6K signaling pathway. Tumour Biol.

[b76] Chua CW, Chiu YT, Yuen HF (2009). Suppression of androgen-independent prostate cancer cell aggressiveness by FTY720: validating Runx2 as a potential antimetastatic drug screening platform. Clin Cancer Res.

[b77] Ushitora Y, Tashiro H, Ogawa T (2009). Suppression of hepatocellular carcinoma recurrence after rat liver transplantation by FTY720, a sphingosine-1-phosphate analog. Transplantation.

[b78] Lee TK, Man K, Ho JW (2005). Significance of the Rac signaling pathway in HCC cell motility: implications for a new therapeutic target. Carcinogenesis.

[b79] Lee TK, Man K, Ho JW (2005). FTY720: a promising agent for treatment of metastatic hepatocellular carcinoma. Clin Cancer Res.

[b80] Shen Y, Cai M, Xia W (2007). FTY720, a synthetic compound from *Isaria sinclairii*, inhibits proliferation and induces apoptosis in pancreatic cancer cells. Cancer Lett.

[b81] LaMontagne K, Littlewood-Evans A, Schnell C (2006). Antagonism of sphingosine-1-phosphate receptors by FTY720 inhibits angiogenesis and tumor vascularization. Cancer Res.

[b82] Kluk MJ, Ryan KP, Wang B (2013). Sphingosine-1-phosphate receptor 1 in classical Hodgkin lymphoma: assessment of expression and role in cell migration. Lab Invest.

[b83] Murali A, Rajalingam K (2014). Small Rho GTPases in the control of cell shape and mobility. Cell Mol Life Sci.

[b84] Steinestel K, Eder S, Schrader AJ (2014). Clinical significance of epithelial-mesenchymal transition. Clin Transl Med.

[b85] Liu H, Gu Y, Yin J (2014). SET-mediated NDRG1 inhibition is involved in acquisition of epithelial-to-mesenchymal transition phenotype and cisplatin resistance in human lung cancer cell. Cell Signal.

[b86] Bergers G, Benjamin LE (2003). Tumorigenesis and the angiogenic switch. Nat Rev Cancer.

[b87] Schmid G, Guba M, Ischenko I (2007). The immunosuppressant FTY720 inhibits tumor angiogenesis *via* the sphingosine 1-phosphate receptor 1. J Cell Biochem.

[b88] Mousseau Y, Mollard S, Faucher-Durand K (2012). Fingolimod potentiates the effects of sunitinib malate in a rat breast cancer model. Breast Cancer Res Treat.

[b89] McGee MC, Hamner JB, Williams RF (2010). Improved intratumoral oxygenation through vascular normalization increases glioma sensitivity to ionizing radiation. Int J Radiat Oncol Biol Phys.

[b90] Huang G, Chen L (2010). Recombinant human endostatin improves anti-tumor efficacy of paclitaxel by normalizing tumor vasculature in Lewis lung carcinoma. J Cancer Res Clin Oncol.

[b91] Soleimani R, Heytens E, Oktay K (2011). Enhancement of neoangiogenesis and follicle survival by sphingosine-1-phosphate in human ovarian tissue xenotransplants. PLoS ONE.

[b92] Li CX, Shao Y, Ng KT (2012). FTY720 suppresses liver tumor metastasis by reducing the population of circulating endothelial progenitor cells. PLoS ONE.

[b93] Diakos CI, Charles KA, McMillan DC (2014). Cancer-related inflammation and treatment effectiveness. Lancet Oncol.

[b94] Hanahan D, Weinberg RA (2011). Hallmarks of cancer: the next generation. Cell.

[b95] Conzett KB, Kolm I, Jelcic I (2011). Melanoma occurring during treatment with fingolimod for multiple sclerosis: a case report. Arch Dermatol.

[b96] Ingwersen J, Aktas O, Kuery P (2012). Fingolimod in multiple sclerosis: mechanisms of action and clinical efficacy. Clin Immunol.

[b97] Lorvik KB, Bogen B, Corthay A (2012). Fingolimod blocks immunosurveillance of myeloma and B-cell lymphoma resulting in cancer development in mice. Blood.

[b98] Omar HA, Chou CC, Berman-Booty LD (2011). Antitumor effects of OSU-2S, a nonimmunosuppressive analogue of FTY720, in hepatocellular carcinoma. Hepatology.

[b99] Mani R, Mao Y, Frissora FW (2015). Tumor antigen ROR1 targeted drug delivery mediated selective leukemic but not normal B-cell cytotoxicity in chronic lymphocytic leukemia. Leukemia.

[b100] Fransson R, McCracken AN, Chen B (2013). Design, synthesis, and anti-leukemic activity of stereochemically defined constrained analogs of FTY720 (Gilenya). ACS Med Chem Lett.

[b101] Tonelli F, Alossaimi M, Natarajan V (2013). The roles of sphingosine kinase 1 and 2 in regulating the metabolome and survival of prostate cancer cells. Biomolecules.

[b102] Tonelli F, Alossaimi M, Williamson L (2013). The sphingosine kinase inhibitor 2-(p-hyroxyanilino)-4-(p-chlorophenyl)thiazole reduces androgen receptor expression *via* an oxidative stress-dependent mechanism. Br J Pharmacol.

[b103] Lim KG, Tonelli F, Li Z (2011). FTY720 analogues as sphingosine kinase 1 inhibitors: enzyme inhibition kinetics, allosterism, proteasomal degradation, and actin rearrangement in MCF-7 breast cancer cells. J Biol Chem.

[b104] Knott K, Kharel Y, Raje MR (2012). Effect of alkyl chain length on sphingosine kinase 2 selectivity. Bioorg Med Chem Lett.

[b105] Lim KG, Sun C, Bittman R (2011). (R)-FTY720 methyl ether is a specific sphingosine kinase 2 inhibitor: effect on sphingosine kinase 2 expression in HEK 293 cells and actin rearrangement and survival of MCF-7 breast cancer cells. Cell Signal.

[b106] Watson DG, Tonelli F, Alossaimi M (2013). The roles of sphingosine kinases 1 and 2 in regulating the Warburg effect in prostate cancer cells. Cell Signal.

[b107] Mao Y, Wang J, Zhao Y (2014). A novel liposomal formulation of FTY720 (fingolimod) for promising enhanced targeted delivery. Nanomedicine.

[b108] Yu B, Mao Y, Yuan Y (2013). Targeted drug delivery and cross-linking induced apoptosis with anti-CD37 based dual-ligand immunoliposomes in B chronic lymphocytic leukemia cells. Biomaterials.

[b109] Pantziarka P, Bouche G, Meheus L (2015). Repurposing drugs in your medicine cabinet: untapped opportunities for cancer therapy?. Future Oncol.

[b110] Cheng X, Bennett RL, Liu X (2013). PKR negatively regulates leukemia progression in association with PP2A activation, Bcl-2 inhibition and increased apoptosis. Blood Cancer J.

[b111] Shen Y, Wang X, Xia W (2008). Antiproliferative and overadditive effects of rapamycin and FTY720 in pancreatic cancer cells *in vitro*. Transplant Proc.

